# To Remind or Not to Remind During Recruitment? An Analysis of an Online Panel in Germany

**DOI:** 10.3389/ijph.2024.1606770

**Published:** 2024-03-20

**Authors:** Bianca Klee, Daniela Costa, Thomas Frese, Anja Knoechelmann, Gabriele Meyer, Thorsten Meyer, Oliver Purschke, Jan Schildmann, Anke Steckelberg, Rafael Mikolajczyk

**Affiliations:** ^1^ Institute for Medical Epidemiology, Biometrics and Informatics, Interdisciplinary Centre for Health Sciences, Medical Faculty of the Martin Luther University Halle-Wittenberg, Halle, Saxony-Anhalt, Germany; ^2^ Institute for General Medicine, Interdisciplinary Centre for Health Sciences, Medical Faculty of the Martin Luther University Halle-Wittenberg, Halle, Saxony-Anhalt, Germany; ^3^ Institute of Medical Sociology, Interdisciplinary Centre for Health Sciences, Medical Faculty of the Martin Luther University Halle-Wittenberg, Halle, Saxony-Anhalt, Germany; ^4^ Institute for Health and Nursing Sciences, Interdisciplinary Centre for Health Sciences, Medical Faculty of the Martin Luther University Halle-Wittenberg, Halle, Saxony-Anhalt, Germany; ^5^ Institute for Rehabilitation Medicine, Interdisciplinary Centre for Health Sciences, Medical Faculty of the Martin Luther University Halle-Wittenberg, Halle, Saxony-Anhalt, Germany; ^6^ Institute for History and Ethics of Medicine, Interdisciplinary Centre for Health Sciences, Medical Faculty of the Martin Luther University Halle-Wittenberg, Halle, Saxony-Anhalt, Germany

**Keywords:** response, participation, online study, bias, web-based survey

## Abstract

**Objective:** To explore the role of reminders in recruiting and maintaining participation in an online panel.

**Methods:** 50,045 individuals from five German federal states were invited by regular mail to participate in the online study “Health-Related Beliefs and Healthcare Experiences in Germany.” Those who did not respond to the first attempt received a postal reminder. Comparisons of sociodemographic characteristics and responses were made between first-attempt respondents and those who enrolled after the second letter.

**Results:** After the initial letter, 2,216 (4.4%, 95%CI: 4.3%–4.6%) registered for the study; after a reminder 1,130 (2.5%, 2.3%–2.6% of those reminded) enrolled. Minor sociodemographic differences were observed between the groups and the content of the responses did not differ. Second-attempt respondents were less likely to participate in subsequent questionnaires: 67.3% of first-attempt vs. 43.3% of second-attempt respondents participated in their fourth survey. Recruitment costs were 79% higher for second-attempt respondents.

**Conclusion:** While reminders increased the number of participants, lower cost-effectiveness and higher attrition of second-attempt respondents support the use of single invitation only for studies with a similar design to ours when the overall participation is low.

## Introduction

Participation in epidemiological studies has sharply declined in the last decades [1, 2]. While studies in the least developed countries can still include high percentages of those initially invited [3], participation in more developed countries is often low nowadays [4]. For example, the UK Biobank study was able to recruit 5.5% of the invited participants using two letters of invitation three weeks apart [5]. The German National Cohort (NAKO) achieved an overall rate of 17% at baseline [6], using telephone calls to potential participants with an available landline after the first invitation and up to three postal reminders for those who could not be contacted by telephone.

When conducting primary data collection, a large sample size implies high power and precision [7, 8]. Another point is to obtain a high level of participation among those who are invited; the effort to achieve this is justified in order to avoid selection bias and improve representativeness [9–11]. However, there is a growing understanding that low participation does not automatically lead to selection bias or compromise the validity of the study [12, 13].

Earlier observations, when participation was still high, indicated that those recruited with more effort (e.g., additional reminders) differed from those recruited earlier and were more similar to ultimate non-respondents [14]. Thus, some have argued that studying late respondents would help to inform about non-respondents, and their inclusion in the sample could help to prevent biased conclusions [15–17]. However, this recommendation is based on findings from the 1980s, when representativeness was associated with 80% participation [18]. Given the current low participation rates, the same reasoning may no longer apply. A first insight is provided by a study with participants from NAKO, where reminders were used to increase participation, and showed that subjects recruited with more effort (two or even three reminders) and those who responded to the initial invitation, did not differ in their characteristics [19].

In addition to considerations related to single assessments, there is also a longitudinal perspective. Although further efforts during recruitment (e.g., through reminder letters, phone calls, and home visits) result in an increase in response proportions [5, 6, 19, 20], studies have indicated that participants recruited with more effort drop out earlier and are therefore of limited benefit for longitudinal studies [10, 18]. These participants may be different from those who remain in the study, but if they are lost, there is not much that can be done to control for this selection. Nonetheless, similar to low response at baseline, low response at follow-ups does not necessarily seem to imply the existence of selection bias [21, 22], but the investment at baseline is lost if follow-up is not continued.

Online studies have emerged in the past two decades as an alternative to traditional paper- or telephone-based data collection, and their use has increased in recent years, particularly during the COVID-19 pandemic [23]. This format presents many advantages but also brings new challenges [24, 25]. While online studies often suffer from an unknown sampling framework, based on volunteers (e.g., when participants are recruited through social media) [26], systematic population-based approaches to recruitment can also be used. To date, it is not clear whether the known strategies, such as repeated postal reminders, also work for online studies.

Using a practical example of an online panel with a population-based recruitment approach, we assessed the effects of sending postal reminders compared to a single postal invitation on the recruited fraction and continued participation.

## Methods

### Study Design

This analysis is based on the population-based study “Health Related Beliefs and Healthcare Experiences in Germany” (HeReCa) [27–30]. The aim of this study is to assess perceptions and experiences regarding health-related topics. Participants receive an email invitation to complete a short online survey (each 10–15 min) three to four times per year. The Ethics Committee of the Medical Faculty of the Martin Luther University Halle-Wittenberg, Germany (No. 2019-044) approved the study, which was conducted in accordance with the Declaration of Helsinki.

### Recruitment of Participants

**FIGURE 1:**
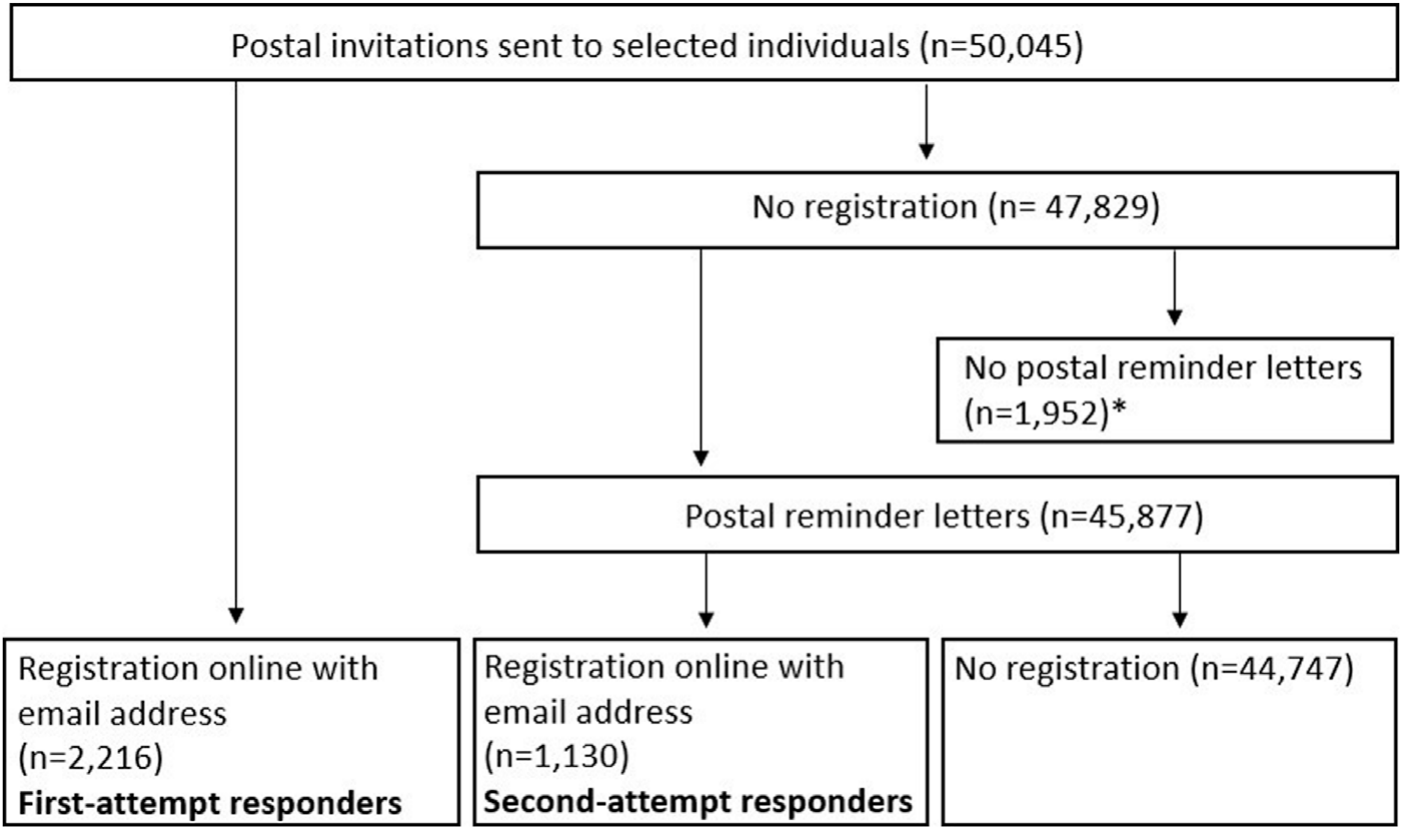
Flowchart of the study population (Health-Related Beliefs and Healthcare Experiences in Germany study, Germany, 2023). * Some individuals actively refused to be contacted and others had letters returned stating that they could not be reached at the provided address. Both groups were excluded from the reminder letters.

We randomly selected 14–15 municipalities (over 5,000 inhabitants) or cities in each of five federal states (Saxony-Anhalt, Berlin, Schleswig-Holstein, Nordrhein-Westfalen, and Baden-Wuerttemberg) in Germany, and asked the relevant resident offices for a random sample of a defined number of persons aged 18–79 (a total of 10,000 per federal state). The municipalities and cities were stratified to represent the distribution of the population density (dense, medium, or sparse) in the given federal state. We mailed invitation letters to the selected residents (Figure 1). These letters contained information about HeReCa and a web link for registration. In order to finalize the registration process, it was crucial to provide the unique code specified in the accompanying letter and an email address. This email address was utilized for subsequent invitations to complete questionnaires, which were accessible via a provided web link. We sent a postal reminder six to twelve months (depending on the federal state) after the first postal invitation to those who had not yet registered and contacted us with a request to be actively removed from the study. Participants provided their informed consent online and did not receive any kind of allowance.

### Data Collection

By March 2023, we had administered twelve different questionnaires in HeReCa. A multi-professional team of health scientists developed questionnaires covering contemporary health issues (e.g., transplantation law in Germany, knowledge about medical rehabilitation, various aspects of COVID-19, and perceptions related to climate change). Participants provided sociodemographic information as part of the first questionnaire. Given that participants were recruited from different federal states over time, some questionnaires were administered in fewer federal states. Starting in April 2021 all participants received the same questionnaires. After an invitation to a specific questionnaire, participants received two reminder emails, one after 2 weeks and another after 1 week, in case they had not completed the survey by then. The online questionnaires remained active for 2–3 weeks after the second reminder email.

### Response

The response was calculated as the ratio of the number of respondents to the number of persons invited to join the study (at baseline) or the number of respondents to a specific questionnaire among those invited to participate in the questionnaire (at follow-up).

### Costs of Recruitment

We calculated the amount of money necessary to recruit the participants. We included the costs of materials (e.g., paper, flyers) and stamps used for mailings, and divided the total cost of the initial and reminder letters by the number of registrations received after the first invitation and reminder, respectively. Staffing and administrative costs, such as fees charged by resident offices, were not included.

### Statistical Analysis

We compared the sociodemographic characteristics of participants recruited by the first invitation and the reminder letter using absolute and relative frequencies with 95% confidence intervals (CI) and means with standard deviation (SD). We also compared the content of the responses between these two groups using Chi-Squared tests, accounting for the false discovery rate (FDR). Finally, we assessed the probability of completing subsequent questionnaires in both groups. R software (Version 4.1.1) was used for all statistical analyses.

## Results

### Comparison of Sociodemographic Characteristics of Participants Recruited by Initial Invitation or Reminder Letter

We sent out 50,045 postal invitations and received 2,216 registrations resulting in a response rate of 4.4% (95%CI: 4.3%–4.6%). These participants will be referred to as “first-attempt respondents” in the following analyses. In total, 45,877 subjects did not reply to the initial invitation within six to twelve months and were reminded about the registration with a follow-up postal letter. This way we recruited an additional 1,130 participants (2.5%, 95%CI: 2.3%–2.6% of those who received a reminder letter; 2.3%, 95%CI: 2.1%–2.4% of those who were initially invited). These participants will be referred to as “second-attempt respondents” in the following analyses (Figure 1). The two groups differed only slightly in sociodemographic characteristics (Table 1). Incomplete responses (missing category) were more common among “second-attempt respondents.” A comparison between the vocational training qualification data of the HeReCa panel and the micro census data in Germany showed a trend toward higher education attainment among HeReCa participants (Supplementary Table S1). We sent the first postal invitations in two federal states (Saxony-Anhalt, Berlin) before the COVID-19 pandemic (November 2019) and in the remaining three federal states (Schleswig-Holstein, Nordrhein-Westfalen, and Baden-Wuerttemberg) during the early phase of the pandemic (between March and May 2020). There was no indication of a higher response during the pandemic (Supplementary Table S2).

**TABLE 1:** Comparison of sociodemographic characteristics between participants who registered for the study after the first invitation letter (first-attempt respondents) and after the reminder invitation letter (second-attempt respondents) (Health-Related Beliefs and Healthcare Experiences in Germany study, Germany, 2023).

	First-attempt respondents N (%)	Second-attempt respondents N (%)
Number of sent postal invitations	50,045 (100)	45,877 (100)
Number of registrations	2,216 (4.4)	1,130 (2.5)
Number of completed sociodemographic questionnaires	2,120 (4.2)	1,074 (2.3)
Age at registration, years		
Mean (SD)	50.8 (15.1)	51.0 (15.5)
18–20	11 (0.5)	0 (0.0)
21–30	242 (11.4)	128 (11.9)
31–40	292 (13.8)	158 (14.7)
41–50	345 (16.3)	145 (13.5)
51–60	534 (25.2)	244 (22.7)
61–70	392 (18.5)	200 (18.6)
71–80	190 (9.0)	100 (9.3)
>80	0 (0.0)	5 (0.5)
Missing	114 (5.4)	94 (8.8)
Sex		
Female participants	1,062 (50.1)	499 (46.5)
Male participants	940 (44.3)	479 (44.6)
Other	3 (0.1)	2 (0.2)
Missing	115 (5.4)	94 (8.8)
Country of birth		
Germany	1,861 (87.8)	909 (84.6)
Other	118 (5.6)	66 (6.1)
Missing	141 (6.7)	99 (9.2)
Marital status		
Married	1,238 (58.4)	587 (54.7)
Unmarried/single	536 (25.3)	251 (23.4)
Divorced	170 (8.0)	82 (7.6)
Widowed	53 (2.5)	46 (4.3)
Married, but living apart	0 (0.0)	11 (1.0)
Missing	123 (5.8)	97 (9.0)
Employment		
Full-time	947 (44.7)	498 (46.9)
Part-time	411 (19.4)	165 (15.4)
Not working	547 (25.8)	260 (24.2)
Not regularly employed	26 (1.2)	6 (0.6)
Other (e.g., parental leave)	66 (3.1)	47 (4.4)
Missing	123 (5.8)	98 (9.1)
Education (according to ISCED 97^32^)		
Low	71 (3.3)	23 (2.1)
Medium	592 (27.9)	321 (29.9)
High	1,225 (57.8)	564 (52.5)
Missing	232 (10.9)	166 (15.5)
Household income in Euro		
<1,250	141 (6.7)	57 (5.3)
1,250- <1750	127 (6.0)	81 (7.5)
1750- <2,250	202 (9.5)	107 (10.0)
2,250- <3,000	322 (15.2)	179 (16.7)
3,000- <4,000	369 (17.4)	164 (15.3)
4,000- <5,000	276 (13.0)	113 (10.5)
>5,000	354 (16.7)	151 (14.1)
I do not want to answer	202 (9.5)	119 (11.1)
Missing	127 (6.0)	103 (9.6)
Number of people in the household		
1	370 (17.5)	186 (17.3)
2	976 (46.0)	486 (45.3)
3	324 (15.3)	145 (13.5)
4	240 (11.3)	121 (11.3)
5	58 (2.7)	20 (1.9)
6	14 (0.7)	10 (0.9)
>6	4 (0.2)	2 (0.2)
Missing	134 (6.3)	104 (9.7)

Given the overall low response, the main cost component was postal letters. The cost per letter was approximately 0.83€ including printing of materials, resulting in recruitment costs of 19€ per first-attempt responder and 34€ per second-attempt responder (+79%).

### Comparison of Response Content Between First- and Second-Attempt Respondents

We analyzed the answers to a questionnaire containing 81 questions about medical care during and before the COVID-19 pandemic. After adjusting for multiple tests using the False Discovery Rate, only one of the items in the questionnaire showed significant differences between the two groups. This question asked whether the participant had any further conditions that required continuous medical treatment, medication use, or a stay in a hospital in the year 2019. Second-attempt respondents showed reduced odds for this condition compared to first-attempt respondents (Odds Ratio = 0.7, 95%CI: 0.5–0.9). The preceding question in the questionnaire listed common diseases (e.g., hypertension, cardiovascular disease) and the participants could indicate whether they had a specific disease. This question was answered similarly in both groups suggesting that the only difference may be random rather than systematic.

### Comparison of First- and Second-Attempt Respondents’ Participation in Subsequent Questionnaires

We selected the second, third, and fourth individual questionnaires for each participant in order to analyze how many participants stayed on the panel over time compared to the number of initial registrations. First, we studied the active withdrawal of consent (Table 2). The loss of participants due to active withdrawal was similar after each subsequent questionnaire and was less than 1 percentage point for the first respondents and approximately 2–3 percentage points for the second-attempt respondents per questionnaire.

**TABLE 2:** Retained fraction of persons (in % related to the persons initially participating by recruitment group), considering only the active withdrawal of study participants (Health-Related Beliefs and Healthcare Experiences in Germany study, Germany, 2023).

	First-attempt respondents proportion (in %) with 95%CI	Second-attempt respondents proportion (in %) with 95%CI
Second questionnaire	99.2 (98.7–99.5)	98.6 (97.7–99.2)
Third questionnaire	98.6 (97.9–99.0)	95.7 (94.3–96.7)
Fourth questionnaire	97.9 (97.2–98.5)	93.5 (91.1–94.9)

**FIGURE 2:**
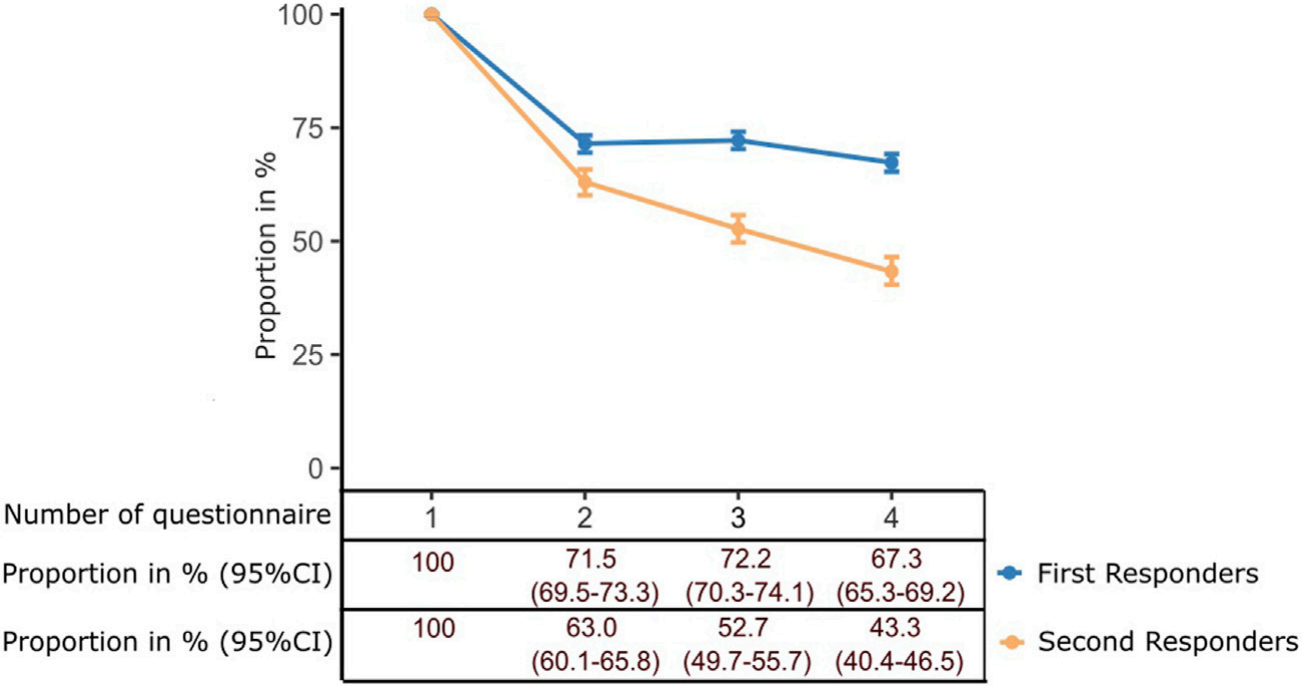
Participation in follow-up questionnaires by recruitment group (points indicate proportion estimates and whiskers the 95% Confidence interval) (Health-Related Beliefs and Healthcare Experiences in Germany study, Germany, 2023).

In contrast, attrition by simply not responding to subsequent questionnaires was much more common among second-attempt respondents compared to first-attempt respondents (Figure 2). After the initial decline from baseline to the first follow-up questionnaire, first-attempt respondents displayed only a slight decline in participation. In contrast, second-attempt respondents showed a more pronounced decline in participation in subsequent questionnaires.

## Discussion

Sending a postal reminder after an online panel invitation resulted in an additional registration of 2.3% of invited individuals, increasing the initial response rate from 4.4% to 6.7% (i.e., +52%). Second-attempt respondents were similar to first-attempt respondents in terms of sociodemographic characteristics. Furthermore, the content of the responses was similar in both groups. Recruitment of second-attempt respondents was 79% more expensive than recruitment of first-attempt respondents (per recruited participant). While active withdrawal in subsequent rounds was only marginally different in both groups, attrition was much higher among those recruited with the reminder.

We did not detect any sociodemographic differences among first- and second-attempt respondents. Clearly, our study was not very large and in larger samples, some differences might appear. Still, the question is whether these differences are relevant. Undoubtedly, some specific groups may have been missed in our study, but it is not clear whether these groups - present in the population - could be recruited, even with more effort. Overall, it seems that the segment of the population accessible for recruitment is limited and within this segment achieving a higher response does not lead to qualitatively different results. Our participants were better educated than the general population, which is in line with other studies. There is clear evidence that participants in a cohort or cross-sectional study are more likely to be female, to have a high socioeconomic status and to have a healthier lifestyle compared to non-respondents [17, 31], and the reminder did not change this distribution in our study.

Our data come from an online study. In recent years, there seems to be a transition from paper-based to web-based surveys, with some studies already reporting higher response rates in web-based surveys than in paper-based surveys [32, 33]. Web-based studies have the advantage of fast and cost-effective data collection, and it has been repeatedly shown that there is no distortion in the results when comparing the two methods [34–36]. Cohort studies with purely digital participation are not yet well established in Germany, but digitization processes and the extensive use of smart devices are becoming more and more integrated into daily life supporting an increasing use of digital data collection in the future. However, recruitment of participants for online studies remains challenging. The majority of online studies do not have a systematic framework for population-based recruitment but rather use advertising and social media. Validation of true participants is often difficult. In Germany, there is no population registry of email addresses that could be used for systematic sampling, therefore we had to resort to a mailed invitation. In the future, residents may potentially have an email address linked to their registration at local government offices, in addition to their home address. Another idea could be to use a hybrid approach that allows for non-systematic recruitment through general advertisement and social media and a systematic population-based sample through postal letters. These two groups could be compared in the analysis for validation. We have applied this recruitment strategy to our digital research platform DigiHero, which currently includes participants from nearly 100,000 households in Germany [37].

Because the HeReCa panel uses only web-based questionnaires, one might expect higher participation among younger participants, but this was not the case. Although evidence is still scarce, two studies showed lower participation for those born 1982–2003 compared to older cohorts in online surveys [38, 39], which is in agreement with our results. This may reflect the increasing internet literacy of older age groups in the population, counterbalancing the lower willingness to participate of younger age groups. In an earlier study, we showed that responses were similar in a population-based panel that allowed paper participation for those who wished, and in an online-only panel [40].

### Strengths and Limitations

The strength of our study is the population-based sampling across five federal states in Germany. Furthermore, in contrast to studies that sent reminders after a short time period, where respondents to the different waves might overlap, we could fully separate the groups that responded to the initial invitation and the reminder. There are also limitations. Our study was conducted only in German, so it is limited to the native population and well-integrated immigrants (in terms of language). The content of our study is general perceptions and considerations related to health topics, so our findings may not be generalizable to investigations on different topics. The topics of the respective questionnaires were announced in the invitation email to complete the questionnaires and topic preferences could affect the decision to fill out a specific questionnaire.

### Conclusion

A postal reminder increased the number of subjects recruited, but those recruited with more effort did not differ substantially in sociodemographic terms from first-attempt respondents. Moreover, first- and second-attempt respondents provided similar responses to the questionnaires presented. Given the higher recruitment costs and higher attrition when using reminders, our results support the use of a single invitation letter in a population-based online study, when participants need to be invited by regular mail.

## Data Availability

The datasets used and/or analyzed during the current study are available from the corresponding author upon reasonable request.

## Abbreviations

NAKO, German National Cohort; HeReCa, Health-Related Beliefs and Healthcare Experiences in Germany; 95% CI, 95% confidence interval; SD, standard deviation; FDR, false discovery rate; ISCED 97, International Standard Classification of Education 1997.
