# Supplementary material for: To Remind or Not to Remind During Recruitment? An Analysis of an Online Panel in Germany
Source: Int J Public Health. 2024 Mar 20;69:1606770. doi: 10.3389/ijph.2024.1606770 (PMC10996063; doi:10.3389/ijph.2024.1606770)
Supplement: Supplementary file 1 [file Table1.docx]

**Supplementary Material**

**Table S1: Comparison of education between micro census data^1^ and HeReCa participants by federal state (Germany, 2023)**

| Highest education obtained | Baden-Wuerttemberg | | Berlin | | Nordrhein- Westfalen | | Saxony Anhalt | | Schleswig-Holstein | |
| --- | --- | --- | --- | --- | --- | --- | --- | --- | --- | --- |
|  | HeReCa | Census | HeReCa | Census | HeReCa | Census | HeReCa | Census | HeReCa | Census |
| Without vocational qualification/in vocational training | 5% | 27% | 5% | 24% | 7% | 30% | 5% | 17% | 5% | 23% |
| Vocational training in the dual system | 25% | 42% | 18% | 37% | 32% | 46% | 34% | 56% | 33% | 52% |
| Technical college degree | 12% | 10% | 11% | 6% | 12% | 7% | 21% | 13% | 14% | 7% |
| Bachelor degree | 6% | 3% | 5% | 5% | 5% | 3% | 3% | 1% | 4% | 2% |
| Master/Diploma degree | 33% | 15% | 41% | 26% | 29% | 13% | 28% | 10% | 30% | 13% |
| PhD degree | 7% | 1% | 7% | 2% | 4% | 1% | 3% | 1% | 4% | 1% |
| No specification | 12% | 0% | 13% | 0% | 11% | 0% | 6% | 0% | 9% | 0% |

^1^ Available from the German Statistical Office: <https://www-genesis.destatis.de/genesis/online?sequenz=tabelleErgebnis&selectionname=12211-9015&transponieren=true#abreadcrumb> (accessed 24^th^ January 2024).
