# Supplementary material for: To Remind or Not to Remind During Recruitment? An Analysis of an Online Panel in Germany
Source: Int J Public Health. 2024 Mar 20;69:1606770. doi: 10.3389/ijph.2024.1606770 (PMC10996063; doi:10.3389/ijph.2024.1606770)
Supplement: Supplementary file 2 [file Table2.docx]

**Supplementary Material**

**Table S2: Comparison of registrations after initial invitations and reminder letters by federal state and age group (Germany, 2023)**

| **Federal State** | **Age Group** | **Number of invitations** | **Month/**  **Year** | **Number of registrations after invitation** | **Response** | **Number of reminders** | **Month/**  **Year** | **Number of registrations after reminder** | **Response** |
| --- | --- | --- | --- | --- | --- | --- | --- | --- | --- |
| Baden-Wuerttemberg | 18-20 | 37 | 05/2020 | 2 | 5% | 35 | 11/2020 | 2 | 6% |
| Baden-Wuerttemberg | 21-30 | 1641 | 05/2020 | 70 | 4% | 1476 | 11/2020 | 30 | 2% |
| Baden-Wuerttemberg | 31-40 | 1775 | 05/2020 | 81 | 5% | 1620 | 11/2020 | 36 | 2% |
| Baden-Wuerttemberg | 41-50 | 1715 | 05/2020 | 93 | 5% | 1578 | 11/2020 | 27 | 2% |
| Baden-Wuerttemberg | 51-60 | 2053 | 05/2020 | 113 | 6% | 1911 | 11/2020 | 53 | 3% |
| Baden-Wuerttemberg | 61-70 | 1674 | 05/2020 | 93 | 6% | 1548 | 11/2020 | 38 | 2% |
| Baden-Wuerttemberg | 71-80 | 1115 | 05/2020 | 43 | 4% | 1048 | 11/2020 | 21 | 2% |
| **Total Baden-Wuerttemberg** | **20-80+** | **10010** | 05/2020 | **495** | **5%** | **9216** | 11/2020 | **207** | **2%** |
| Berlin | 18-20 | 40 | 11/2019 | 2 | 5% | 37 | 05/2020 | 0 | 0% |
| Berlin | 21-30 | 1590 | 11/2019 | 41 | 3% | 1355 | 05/2020 | 32 | 2% |
| Berlin | 31-40 | 2232 | 11/2019 | 79 | 4% | 1950 | 05/2020 | 43 | 2% |
| Berlin | 41-50 | 1621 | 11/2019 | 59 | 4% | 1478 | 05/2020 | 47 | 3% |
| Berlin | 51-60 | 1947 | 11/2019 | 108 | 6% | 1751 | 05/2020 | 56 | 3% |
| Berlin | 61-70 | 1444 | 11/2019 | 76 | 5% | 1323 | 05/2020 | 48 | 4% |
| Berlin | 71-80 | 1126 | 11/2019 | 41 | 4% | 1044 | 05/2020 | 23 | 2% |
| **Total Berlin** | **20-80+** | **10000** | 11/2019 | **406** | **4%** | **8938** | 05/2020 | **249** | **3%** |
| Nordrhein-Westfalen | 18-20 | 79 | 05/2020 | 4 | 5% | 72 | 12/2020 | 0 | 0% |
| Nordrhein-Westfalen | 21-30 | 1699 | 05/2020 | 70 | 4% | 1518 | 12/2020 | 36 | 2% |
| Nordrhein-Westfalen | 31-40 | 1599 | 05/2020 | 52 | 3% | 1472 | 12/2020 | 34 | 2% |
| Nordrhein-Westfalen | 41-50 | 1706 | 05/2020 | 73 | 4% | 1584 | 12/2020 | 42 | 3% |
| Nordrhein-Westfalen | 51-60 | 2079 | 05/2020 | 108 | 5% | 1927 | 12/2020 | 45 | 2% |
| Nordrhein-Westfalen | 61-70 | 1687 | 05/2020 | 77 | 5% | 1572 | 12/2020 | 36 | 2% |
| Nordrhein-Westfalen | 71-80 | 1144 | 05/2020 | 35 | 3% | 1083 | 12/2020 | 16 | 1% |
| Nordrhein-Westfalen | NA | 2 | 05/2020 | 0 | - | 2 | 12/2020 | 0 | - |
| **Total Nordrhein-Westfalen** | **20-80+** | **9993** | 05/2020 | **419** | **4%** | **9230** | 12/2020 | **209** | **2%** |
| Saxony-Anhalt | 18-20 | 24 | 11/2019 | 0 | 0% | 23 | 11/2020 | 1 | 4% |
| Saxony-Anhalt | 21-30 | 1084 | 11/2019 | 41 | 4% | 969 | 11/2020 | 15 | 2% |
| Saxony-Anhalt | 31-40 | 1621 | 11/2019 | 44 | 3% | 1525 | 11/2020 | 40 | 3% |
| Saxony-Anhalt | 41-50 | 1577 | 11/2019 | 82 | 5% | 1463 | 11/2020 | 36 | 2% |
| Saxony-Anhalt | 51-60 | 2246 | 11/2019 | 117 | 5% | 2095 | 11/2020 | 62 | 3% |
| Saxony-Anhalt | 61-70 | 2067 | 11/2019 | 85 | 4% | 1941 | 11/2020 | 59 | 3% |
| Saxony-Anhalt | 71-80 | 1401 | 11/2019 | 31 | 2% | 1333 | 11/2020 | 29 | 2% |
| **Total Saxony-Anhalt** | **20-80+** | **10020** | 11/2019 | **400** | **4%** | **9349** | 11/2020 | **242** | **3%** |
| Schleswig-Holstein | 18-20 | 27 | 03/2020 | 1 | 4% | 26 | 12/2020 | 0 | 0% |
| Schleswig-Holstein | 21-30 | 1482 | 03/2020 | 54 | 4% | 1292 | 12/2020 | 30 | 2% |
| Schleswig-Holstein | 31-40 | 1572 | 03/2020 | 66 | 4% | 1421 | 12/2020 | 23 | 2% |
| Schleswig-Holstein | 41-50 | 1677 | 03/2020 | 79 | 5% | 1543 | 12/2020 | 32 | 2% |
| Schleswig-Holstein | 51-60 | 2346 | 03/2020 | 135 | 6% | 2159 | 12/2020 | 72 | 3% |
| Schleswig-Holstein | 61-70 | 1654 | 03/2020 | 101 | 6% | 1530 | 12/2020 | 43 | 3% |
| Schleswig-Holstein | 71-80 | 1262 | 03/2020 | 60 | 5% | 1173 | 12/2020 | 23 | 2% |
| **Total Schleswig-Holstein** | **20-80+** | **10020** |  | **496** | **5%** | **9144** | 12/2020 | **223** | **2%** |
